# Quantitative Analysis of Hepatitis C NS5A Viral Protein Dynamics on the ER Surface

**DOI:** 10.3390/v10010028

**Published:** 2018-01-08

**Authors:** Markus M. Knodel, Arne Nägel, Sebastian Reiter, Andreas Vogel, Paul Targett-Adams, John McLauchlan, Eva Herrmann, Gabriel Wittum

**Affiliations:** 1 Goethe Center for Scientific Computing (G-CSC), Goethe Universität Frankfurt, Kettenhofweg 139, 60325 Frankfurt am Main, Germany; naegel@gcsc.uni-frankfurt.de (A.N.); sreiter@gcsc.uni-frankfurt.de (S.R.); avogel@gcsc.uni-frankfurt.de (A.V.); wittum@gcsc.uni-frankfurt.de (G.W.); 2 Ruhr-Universität Bochum, High Performance Computing in the Engineering Sciences, Universitätsstrasse 150, 44801 Bochum, Germany; 3 Medivir AB, Department of Biology, Huddinge 141 22, Sweden; Paul.Targett-Adams@medivir.com; 4 MRC-University of Glasgow Centre for Virus Research, 464 Bearsden Road, Glasgow G61 1QH, UK; john.mclauchlan@glasgow.ac.uk; 5 Department of Medicine, Institute for Biostatistics and Mathematic Modeling, Goethe Universität Frankfurt, Theodor-Stern-Kai 7, 60590 Frankfurt am Main, Germany; herrmann@med.uni-frankfurt.de; 6 Applied Mathematics and Computational Science, Computer, Electrical and Mathematical Science and Engineering Division, King Abdullah University of Science and Technology, KAUST, Thuwal 23955, Saudi Arabia

**Keywords:** computational virology, hepatitis C virus (HCV), viral dynamics, within-host viral modelling, parameter estimation, 3D spatio-temporal resolved mathematical models, realistic geometries, (surface) partial differential equations, Finite Volumes, massively parallel multigrid solvers

## Abstract

Exploring biophysical properties of virus-encoded components and their requirement for virus replication is an exciting new area of interdisciplinary virological research. To date, spatial resolution has only rarely been analyzed in computational/biophysical descriptions of virus replication dynamics. However, it is widely acknowledged that intracellular spatial dependence is a crucial component of virus life cycles. The hepatitis C virus-encoded NS5A protein is an endoplasmatic reticulum (ER)-anchored viral protein and an essential component of the virus replication machinery. Therefore, we simulate NS5A dynamics on realistic reconstructed, curved ER surfaces by means of surface partial differential equations (sPDE) upon unstructured grids. We match the in silico NS5A diffusion constant such that the NS5A sPDE simulation data reproduce experimental NS5A fluorescence recovery after photobleaching (FRAP) time series data. This parameter estimation yields the NS5A diffusion constant. Such parameters are needed for spatial models of HCV dynamics, which we are developing in parallel but remain qualitative at this stage. Thus, our present study likely provides the first quantitative biophysical description of the movement of a viral component. Our spatio-temporal resolved ansatz paves new ways for understanding intricate spatial-defined processes central to specfic aspects of virus life cycles.

## 1. Introduction

When a virus hijacks host cells of the body, its aim is to subvert cellular metabolism in order to create new virus progeny, which will infect surrounding cells and disseminate the infection. For the host, such events can cause different degrees of cell stress and damage according to the cytopathogenicity of the virus. Hepatitis C virus (HCV) [1] belongs to the plus stranded RNA (ss(+)RNA) viruses [2,3] such as Dengue and Yellow fever viruses (DNFV and YFV) [2,4]; these latter 2 viruses are poised to create increased health problems in the US and Europe over subsequent years; largely driven by climatic changes favoring geographic expansion of their mosquito vector. However, infection by HCV is a current global pandemic and liver disease such as cirrhosis, which is associated with chronic infection by the virus, is the main reason for liver transplantations in Western countries.

Spatial dependence is a crucial factor in the process all viruses use in order to replicate [2,4,5,6,7,8,9,10,11,12,13,14,15,16,17,18,19,20]; many viruses rearrange cellular membranes, or create inclusion bodies, to create specialized regions within the host cell designed to replicate viral nucleic acid and assemble progeny. For HCV, replication is believed to occur in specialized compartments within virus-infected cells, termed membranous webs [1,6,9]. The membranous webs are derived from altered regions of an interconnected intracellular membrane network called the Endoplasmic Reticulum (ER) [21]; the HCV-modified structure is termed the membranous web due to its appearance when viewed by electron microscopy [5,6,9]. Formation, maintenance, regulation, and turnover of membranous webs is likely driven both by interactions between virus-encoded components (virus proteins and RNA), and those with host proteins/organelles. This delicate balance of interactions is likely a dynamic process occurring in 3D that is both difficult to capture experimentally and conceptually visualize [9].

Exploring the biophysics of viral replication mechanisms through cross-discipline work i.e., application of physics-based solutions to understand biology-based data is a highly relevant goal. Previous modeling work at an (intra)cellular level using HCV has focused upon ordinary differential equation (ODE) compartment models, cf. e.g., [22,23,24,25,26].

To our best knowledge, spatial resolution is an aspect that has only been rarely appreciated in biophysical modeling simulations approaches of virus dynamics to date. For a detailed discussion, we refer to our former paper [27]. In the previous paper [27], we developed spatio-temporal resolved (surface) partial differential equation (PDE/sPDE) reaction-diffusion models of the HCV viral RNA (vRNA) replication cycle. Even though these models were computed upon realistic reconstructed cell geometries, they suffered from a lack of experimentally validated parameters. For example, the diffusion constants for the agents of the vRNA cycle, namely for the vRNA and the non structural viral proteins (NSPs), which are responsible for the replication of the vRNA were not known.

The HCV-encoded NS5A protein [6,28,29] belongs to the class of NSPs. NS5A is an essential component of HCV replication and probably contributes many functions that the virus is dependent upon to replicate its RNA and assemble its progeny [1,6,28,30,31]. Research has revealed a substantial spatial facet of NS5A function and particular biophysical characteristics of the protein arise from its anchoring to the 3D embedded curved 2D ER manifold [1,2,5,6]. Inhibition of NS5A functions by small molecule-based inhibitors of the protein [28,30] lead to spatial redistribution of NS5A [7] and also change its mobility characteristics [8,10]. Increasing our knowledge of the properties and functions of the HCV NS5A protein may reveal global processes that ss(+)RNA viruses employ to replicate their genome and, therefore, may ultimately help to identify mechanisms suitable for intervention to enable discovery of broad-spectrum inhibitors.

Therefore, in this study, we present a quantitative, spatially resolved analysis of the NS5A movement dynamics. For this purpose, we combine and compare experimental fluorescence recovery after photobleaching (FRAP) [32,33] time series (TMS) data [34] with mathematical simulations of a model probing a diffusion process of the NS5A protein on realistic reconstructed ER surfaces [5] by means of surface partial differential equations (sPDE). The sPDEs were solved with advanced numerical techniques upon large unstructured grids representing the ER surface [35]. Hence, we present the estimation of the biophysical meaningful NS5A diffusion constant based on the comparison of experimental and simulation data.

To this end, data presented in the present study are complementary to our former paper [27], which demonstrated the development of qualitative spatio-temporal resolved diffusion-reaction models of the vRNA replication cycle of HCV. These models remained qualitative at this stage due to a lack of experimentally validated parameters [27]. Therefore, the present paper demonstrates the estimation of a parameter needed for spatio-temporal resolved diffusion-reaction models of the vRNA replication cycle of HCV. Even though we estimate only one parameter that is needed for such models, our study shows that the estimation of such parameters is a highly nontrivial task. This tasks asks for the combination of advanced experiments and simulations. Future studies are needed to estimate the diffusion constants of all agents entering models of HCV replication. These studies will ask for further experimental studies which have to been combined with simulations in the manner we present here.

Hence, the detailed spatially-resolved understanding of the NS5A dynamics paves a way to advanced quantitative spatially-resolved understanding of HCV replication dynamics. Such a novel approach forms the basis of subsequent approaches extended initially to the other components of the HCV vRNA replication cycle and later, applied to other virus systems. Our approach may be used as first step of computational-based quantitative research in virology using advanced spatio-temporal resolved modeling and simulations techniques. To our best knowledge, the approach to apply highly precise numerical methods upon realistic geometric setups is a technique that has not yet been applied within the computational virology field.

## 2. Materials and Methods

The in silico description of the movement of NS5A is built on three basic components:

- Experimental data for the dynamics: FRAP time series data [34] recorded the intracellular dynamics of NS5A movement [10] at perinuclear zones. These data report the dynamics of NS5A under a variety of biological conditions. Namely we consider two different cell types explained in detail below.
- Geometric setup: We use previously published confocal microscopic microscopy z-stack data [5] of cells labeled with ER markers which allow for reconstructions of realistic ER surfaces. These fine level data provide the geometric constraints for NS5A movement.
- A model and corresponding simulations: Our previous model of NS5A dynamics [35] has not been adapted to biological data so far. In this study, we perform simulations using an extended version of the model and fit the simulation parameters in order to match the experimental data.

In the below, we describe these components in greater detail i.e. the FRAP time series experimental data, realistic ER geometry reconstructions, and we provide a short summary on NS5A movement properties that underpin the model we describe as basis for the sPDE simulations of NS5A dynamics (introduced by us recently [35]).

### 2.1. FRAP Experiments—Basics

FRAP experiments [32,33] rely on the bleaching and refilling of a region within a cell corresponding to the intracellular location of a fluorescing component of interest, in this case, NS5A. The protein is distributed (and fluoresces) homogeneously at the beginning of the experiment. In a first step, a strong laser beam deletes the fluorescence in a defined region; the cylindrical FRAP region of interest (ROI) F. Subsequently, fluorescence recovers due to the influx of surrounding proteins from the surrounding unbleached region U. Measuring the intensity increase in F by a repetitive application of a second (soft) laser beam, allows to track the movement dynamics of the labelled NS5A proteins.

### 2.2. Expermimental Data and Cell types

FRAP experiments were previously conducted, cf. [34]. The 20 time series published in this paper [34] were divided into two groups of in vitro cell types, namely NS5A/Alone and NS5A/OtherNSPs [34,36,37], with 10 time series for each of the groups.

“NS5A/Alone” are Huh7 cells transfected with a DNA construct that encodes a NS5A-GFP fusion protein, but no other virus proteins are present. “NS5A/OtherNSPs” are Huh7 cells that contain a HCV genotype 2a subgenomic that encodes a NS5A-GFP fusion protein together with HCV non-structural proteins NS3, NS4A, NS4B, and NS5B. Thus; NS5A/OtherNSPs cells constitutively replicate HCV RNA but do not make virus since they don’t produce the virus structural proteins (SPs). They do authentically replicate the virus RNA synthesis machinery though. In NS5A/Alone cells, NS5A-GFP is more mobile because it is likely not sequestered into membranous web regions to the same degree as it is in NS5A/OtherNSPs cells. NS5A/Alone cells allow the investigation of transport processes for less restricted NS5A-GFP. In contrast, NS5A/OtherNSPs cells have NS5A-GFP expressed as part of NS3-5B where NS5A diffusion is more restricted to replication sites.

### 2.3. NS5A Movement Properties

NS5A anchors directly to the ER surface after its cleavage from the polyprotein [7,28,34] (like all HCV NSPs [1]). Hence its movement is restricted to the ER surface and/or membranes derived from this organelle. As a first approximation, we may assume that the movement of this “free” NS5A can be modeled as diffusive movement. However, a proportion of NS5A can subsequently cluster to form vesicles, such as double membrane vesicles (DMVs) [6]. These vesicular structures contribute to the design of the membranous web. To our best knowledge, the relationship between the subpopulations of NS5A that cluster to form DMVs and NS5A that remains free is not yet understood. This applies equally to the NS5A/Alone data where only NS5A is expressed alone, and also for the NS5A that is expressed together with other NSPs, i.e., the NS5A/OtherNSPs cell type. Thus, the degree at which NS5A is associated with DMVs may be different for the two populations of NS5A we describe in the present study i.e., NS5A/Alone compared to NS5A/OtherNSPs.

At peripheral cell regions, the DMV clusters have a tendency to show random walk properties, i.e., to “jump around” [8,9,10,38]. The peripheral NS5A foci (which are putative replication complexes) are frequently highly motile and capable of rapid long-range traffic. Hence, advanced models of NS5A will need to incorporate and addresses the motile populations of NS5A foci, for which particle tracking analysis will be an important evaluation tool, cf, e.g., [39].

However, this “jumping” movement characteristics of NS5A clusters does not appear at perinuclear regions. Indeed, FRAP analysis of intense perinuclear NS5A foci have revealed a relatively static internal architecture [9,34]. Since the FRAP experiments of NS5A [34] (which are the experimental basis for this study) were performed at perinuclear regions, we did not take into account the highly motile subpopulations of DMVs at peripheral subcellular locations during our modeling. Therefore, our present study focuses upon NS5A properties within perinuclear regions of the cell. Extension of the model to include peripheral NS5A properties [8,9,10,38] will be the subject of forthcoming analyses and will require particle tracking analysis and single particle movement to complement our present diffusion/continuum model based framework.

### 2.4. Modeling FRAP Experiments

First FRAP-based approaches were performed about 40 years ago by e.g., Soumpasis [40] and Axelrod et al. [41]. These analyses were based upon the assumption of pure 1D/2D processes, i.e., of mobility which takes place unrestricted within the 2D plane such that in many cases, the models can be reduced to 1D cases, i.e., exponential descriptions allow for solving the models. These basic assumptions are also a central part of more recent approaches for the modeling of FRAP experiments, cf. e.g., [42,43,44,45,46]. and also for similar modeling approaches for other fluorescence experiments like FLIP (fluorescence loss in photobleaching) [47].

Our aim is to model FRAP experiments of the NS5A protein. Diffusive movement of NS5A is restricted to the ER surface while a portion of NS5A clusters to DMV complexes, which likely originate from the ER surface. The scope of this study is to reproduce the experimental time series from data previously published, cf. [34]. These data were produced within perinuclear regions where DMV complex traffic is not a common phenomenon.

We start deriving a transport model for NS5A on the ER surface for a given configuration E (i.e., ER geometry). In the model, we assume that it is reasonable to distinguish between two groups of NS5A molecules:

Let $c^{\left(-\right)}$ and $c^{\left(+\right)}$ denote the surface concentrations of NS5A occuring freely (−) (i.e., not clustering to DMVs or web regions) and in clustered form (+) (i.e., accumulated to DMVs respectively web regions) respectively. Note, that this definition tacitly assumes that it is reasonable to average over a certain area, which is large compared to the representative size of a cluster of molecules for either of the two types. Moreover, it is assumed, that transport of the two NS5A species is due to diffusive transport along the ER, i.e., in E the equations

(1) $$\frac{d}{dt}c^{\left(+\right)}-D^{+}\Delta_{\left(T\right)}c^{\left(+\right)}=k_{\mathrm{on}}\;c^{\left(-\right)}-k_{\mathrm{off}}\;c^{\left(+\right)}$$

(2) $$\frac{d}{dt}c^{\left(-\right)}-D^{-}\Delta_{\left(T\right)}c^{\left(-\right)}=-k_{\mathrm{on}}\;c^{\left(-\right)}+k_{\mathrm{off}}\;c^{\left(+\right)}$$

hold. Here $\Delta_{\left(T\right)}$ denotes the Laplace-Beltrami-Operator, which is the projection of the Laplace operator to the tangential space of the two dimensional ER-hypersurface E that is embedded into $R^{3}$ [48]. The right hand side is a first order transformation model for mass transfer between both (±)-species. This model is further simplified as follows: (i) transport of the (−)-type is much faster than of the (+)-type, and (ii) the characteristic time of the reactions is much faster than the characteristic time for (diffusive) transport, i.e.,

(3) $$k_{\mathrm{on}}^{-1},k_{\mathrm{off}}^{-1}\ll \frac{\lambda^{2}}{D^{-}}\ll \frac{\lambda^{2}}{D^{+}}$$

Here, λ is the characteristic length for the experiment (i.e., the diameter of the blank spot generated by the laser beam in the FRAP experiment). Under these assumptions, we replace (1) by the quasi-steady-state equilibrium

(4) $$k_{\mathrm{on}}\;c^{\left(-\right)}=k_{\mathrm{off}}\;c^{\left(+\right)},$$

and obtain a reduced version for (2):

(5) $$\left(1+\frac{k_{\mathrm{on}}}{k_{\mathrm{off}}}\right)\frac{d}{dt}c^{\left(-\right)}-D^{-}\Delta_{\left(T\right)}c^{\left(-\right)}=0$$

Note that this can formally deduced by summing up (1) and (2) and approximating $D^{+}\approx 0$ due to (3). Introducing the effective concentration

(6) $$c_{\mathrm{ns}5a}:=\left(1+\frac{k_{\mathrm{on}}}{k_{\mathrm{off}}}\right)c^{\left(-\right)}=c^{\left(-\right)}+c^{\left(+\right)},$$

and the (observed) effective diffusion coefficient

(7) $$D_{\mathrm{ns}5a}:=D^{-}/\left(1+\frac{k_{\mathrm{on}}}{k_{\mathrm{off}}}\right)$$

yields the central sPDE for the dynamics of NS5A fluorescence expressed in terms of the intensity density

$$i_{\mathrm{ns}5a}=i_{\mathrm{ns}5a}\left(t,\vec{x}\right)\propto c_{\mathrm{ns}5a}\left(t,\vec{x}\right)$$

on the ER surface domain:

(8) $$\frac{d}{dt}i_{\mathrm{ns}5a}-D_{\mathrm{ns}5a}\Delta_{\left(T\right)}i_{\mathrm{ns}5a}=-{\left.\left[r_{p}i_{\mathrm{ns}5a}\right]\right|}_{F},\;\forall \vec{x}\in E=F\cup U.$$

This is the “master equation” we use for the simulation of the spatially resolved dynamics of the data. In contrast to (5), this equation includes the intensity reduction caused by the intensity measurement with the soft laser as a “pseudo reaction” by a constant $r_{p}$. This effect is discussed in greater detail in the forthcoming Section 2.5. Note, that the constant $r_{p}$ does not correspond to a degradation of NS5A itself. This occurs at a much bigger time scale and is not included in the model.

Note that due to the presence of two different cell types, our framework models two different concentrations for each one of these cell types. Whenever it is important to distinguish, we indicate this by an addition index. We write $c_{A}^{\left(\pm \right)}$ and $i_{\mathrm{ns}5a}^{A}$ for concentrations and intensities of cell type NS5A/Alone, and $c_{N}^{\left(\pm \right)}$ and $i_{\mathrm{ns}5a}^{N}$ for the same quantities when referring to cell type NS5A/OtherNSPs. Likewise, we later derive diffusion constants $D_{\mathrm{ns}5a}^{A}$, $D_{\mathrm{ns}5a}^{N}$ and measurement induced intensity reduction rates $r_{p}^{A}$, $r_{p}^{N}$ for both cell types. The additional indices for the cell lines will be suppressed if the forthcoming statements are valid for both cell types.

In order to match the experimental setup, initial conditions for (8) are provided independently for bleached and unbleached zones/subdomains respectively. (The upper indices refer by now to the subdomains and not to the cell type, the cell type index is suppressed as explained before, since the explanations are valid for both cell types).

(9) $$i_{\mathrm{ns}5a}\left(t_{0},\vec{x}\right)=\left\{\begin{array}{cc} i_{0}^{F}, & \forall \vec{x}\in F,\\ i_{0}^{U}, & \forall \vec{x}\in U. \end{array}\right.$$

Due to the bleaching of the FRAP ROI with the strong laser at the beginning of the experiment, the intensity of the FRAP region is much smaller than of the surrounding unbleached region, $i_{0}^{F}\ll i_{0}^{U}$. The initial values of the simulations, $i_{0}^{F}$ and $i_{0}^{U}$, are determined by experimental values according to $i_{0}^{F}=I_{0}^{F}/I_{0}$ and $i_{0}^{U}=I_{0}^{C}/I_{0}$. Here, $I_{0}$ denotes the intensity in the FRAP region before bleaching, $I^{F}\left(t_{0}-\epsilon \right)$, where 0<ϵ→0. After bleaching, the intensities $I^{F}\left(t_{0}\right)$ and $I^{U}\left(t_{0}\right)$ are determined independently. The value for $I^{U}\left(t_{0}\right)$ is determined in an unbleached control region C⊂U. In this region, intensity is continuously monitored by independent application of a soft laser. The control region C is located in large distance from F , such that the mutual influence is negligible. All values are determined after subtraction of the background noise.

The sPDE (8) computations were performed with UG4 [49,50,51] based on Finite Volumes discretisations [52,53,54] and massively parallel multigrid solvers [55,56]. (UG has been used successfully within various areas of computational physics [57,58] and biophysics, namely computational neuroscience [59,60,61,62] and computational pharmacology [63,64]). For technical details concerning the discretization methods and the massively parallel multigrid solvers, we refer to our former paper [27].

### 2.5. Pseudo Reaction Constant Fit

The application of the soft laser induces an intensity decay, which is reflected by the constant $r_{p}$ in (8). Its value is determined by the signal decay in the (unbleached) control ROI C, assuming the following ODE:

(10) $$\begin{array}{cccc} & \frac{d}{dt}i_{c}\left(t\right) & = & -r_{p}i_{c}\left(t\right)\\ ⟹ & i_{c}\left(t\right) & = & i_{0}\exp\left(-r_{p}\left(t-t_{0}\right)\right). \end{array}$$

Here, $i_{0}$ and $r_{p}$ were determined for each one of the 20 TMS and averaged for both cell types separately by fitting to the corresponding experimental values [34].

**Figure 1:**
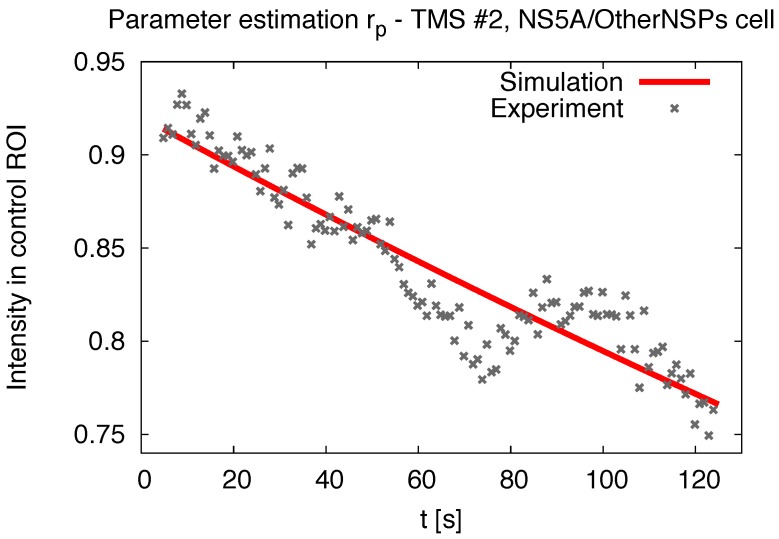
Intensity changes in unbleached control region C according to Equation (10): Experimental data for time series (TMS) #2 (NS5A/OtherNSPs) and the corresponding fit. The determination of the exponential decay rate of the signal itself (far away from the FRAP region, where no diffusion takes place) allows for a more precise evaluation of the diffusion constant.

**Figure 2:**
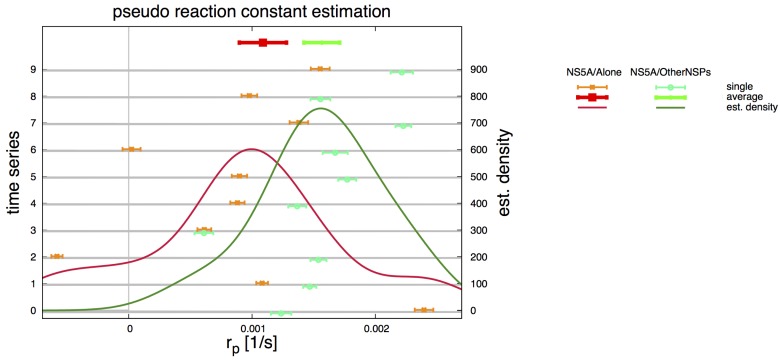
Averages for pseudo reaction rate $r_{p}$—analyzed separately for NS5A/Alone (red—$r_{p}^{A}$) and NS5A/OtherNSPs (green—$r_{p}^{N}$). Thin points and error bars correspond to estimated values for $r_{p}$ (shown on x axis) for a single TMS (indicated on the left y axis). Aggregating over all TMS yields distributions (continuous lines, scale shown on right y axis). Thick symbols (shown on top) correspond to the averaged values ${\bar{r}}_{p}$ reported in Section 2.5. The final averaged values ${\bar{r}}_{p}^{A}$, ${\bar{r}}_{p}^{N}$ enter the diffusion equation of NS5A on the ER surface for the corresponding TMS of the two cell lines.

Figure 1 provides an example of the solution of (10) fitted to the experimental values of the control ROI for one particular time series. Aggregating these results for both cell types yields the distributions shown in Figure 2. (The result for one TMS (NS5A/Alone #2) yielding a negative $r_{p}$ was discarded due to an assumed measurement error. Note: Counting of time series starts from 0 ranging to 9, hence e.g., the 10th TMS has index # 9). The stochastic distributions and averages were computed with standard algorithms [65] using R [66].

The data shows that the values for $r_{p}$ are not correlated and can be distinguished. The reduction rate for NS5A/OtherNSPs cells is higher than for NS5A/Alone:

(11) $$\begin{array}{cccc} {\bar{r}}_{p}^{A} & = & 0.001089\pm 0.000191\;s^{-1} & \;\mathrm{for}\;\mathrm{NS}5A/\mathrm{Alone}\;\mathrm{cells},\;\mathrm{and}\;\\ {\bar{r}}_{p}^{N} & = & 0.001566\pm 0.000145\;s^{-1} & \;\mathrm{for}\;\mathrm{NS}5A/\mathrm{OtherNSPs}\;\mathrm{cells}.\; \end{array}$$

### 2.6. ER Geometry Reconstruction

Our aim was to simulate NS5A diffusion upon real cell geometries. 20 NS5A FRAP time series from a former study [34] are the experimental basis of this study. These 20 time series reflect the NS5A dynamics. Each time series corresponds to one experiment. Each experiment was performed with one in vitro cell. This means that each time series corresponds with one special cell geometry. Each time series corresponds to one cell structure. We wanted to solve sPDE (8) upon realistic reconstructed ER surfaces. The computational domain E for sPDE (8) corresponds to the ER surfaces for each one of the 20 time series [34]. We wanted to reconstruct the cell structure of each time series. On this way, 20 ER geometries would have arisen.

However, there is a general technical limitation: An in vitro cell can be used *either* for FRAP experiments, *or* for staining purposes. *Simultaneous* staining of compartments of a cell *and* recording of FRAP time series at the same cell is not possible. This means, one can use the cell *either* to record time series. *Or* one can use the cell to stain special compartments like the ER. Such staining is the basis for reconstructions. However, a cell used to perform a FRAP time series cannot be stained. Compartments which are not stained cannot be reconstructed. Since it is not possible to use the same cell to record dynamics *and* to stain compartments, the cell geometry of a FRAP time series cell cannot be reconstructed. To this end, the ER geometry of a FRAP time series cannot be used as basis for FRAP simulations. There was no possibility to reconstruct the ER geometries which correspond to the cells of the 20 FRAP experiments. There was no possibility to evaluate sPDE (8) upon one of the cell geometries where the experimental time series [34] were recorded.

**Figure 3:**
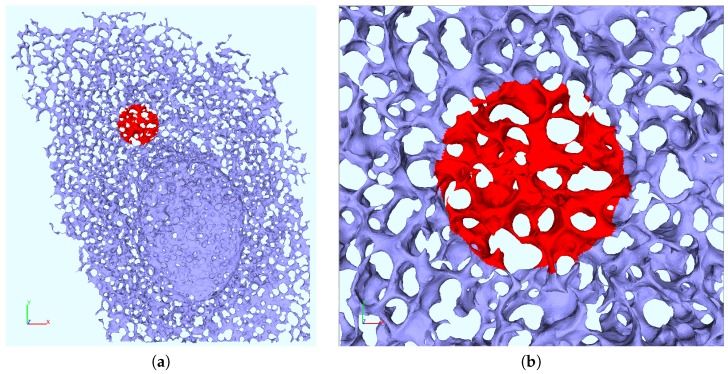
Surface mesh of reconstructed ER geometry $E_{1}$. (**a**) Computational domain used for the simulations of the FRAP experiments of NS5A on (intra)cellular level. Dark blue: unbleached region U, Red: FRAP region F used for bleaching (covering a surface of 38 μm${}^{2}$ in the 2D projection plane as in experiment); cf. Section 2.1 and Section 2.6. (**b**) Magnification around FRAP ROI F.

Despite all these adversities, our aim was to perform our simulations upon realistic geometric environments. Therefore, we reconstructed 5 realistic ER surfaces [35] using NeuRA2.3 [67,68] based on (with Huygens [69] deblured) z-stacks from another former study [5]. For each ER geometry, we were probing for 2 different exemplary FRAP ROIs (selected manually) with a size of 38 ${\left(\mu m\right)}^{2}$ as in the FRAP experiments [34]. Figure 3 shows an example of such a geometric setup. We performed various tests to ensure that the choice of these geometries is justified, cf. Section 2.7 and Section 3.3.

The basic geometry details (like vertex numbers of the unstructured triangular grids—about $10^{6}$ at base level), discretization and solver properties were presented in our former paper [35] as basis for extended refinement stability checks of single sPDE evaluations (using heuristic values for the diffusion constant). The Appendix B and Appendix C give additional information on the geometries. (ER geometry indexing starts from 1 ranging to 10).

### 2.7. Comparing Experiment and Simulation

We had to estimate the NS5A diffusion constant for two different experimental scenarios, namely NS5A/Alone cells and NS5A/OtherNSPs cells, cf. Section 2.2. For the comparison with experimental FRAP ROI intensity (cf. Section 2.4), we define the integrated normalized luminosity

(12) $$I\left(t\right)=\frac{\int_{F}i_{\mathrm{ns}5a}\left(t,\vec{x}\right)d\vec{x}}{\int_{F}d\vec{x}}$$

obtained from the simulations. $i_{\mathrm{ns}5a}$ is computed as solution of the sPDE (8) for each one of the following combinations: Combining 10 reconstructed geometric setups with 20 experimental time series, yields a total of 200 combinations. In order to find optimal values of $D_{\mathrm{ns}5a}$ for each one of the 200 combinations of time series—geometric setup, we used the Gauss-Newton procedure [70].

The averaging for final diffusion constant values was performed over the single estimated values for the combination of all geometric setups for each cell type over its 10 respective time series. All averaging procedures and all distribution computations elaborated in this study were performed with standard algorithms [65] implemented within the program code R [66].

## 3. Results

### 3.1. Realistic Simulation of FRAP Experiments

**Figure 4:**
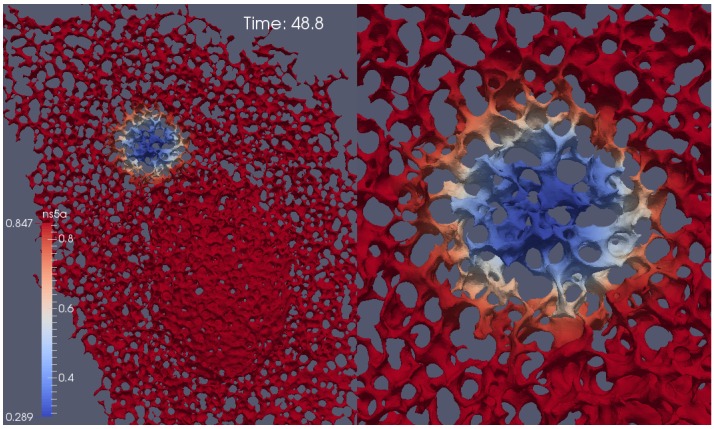
Simulation of NS5A concentration at the ER surface during a FRAP experiment, screenshot of supplemental movie “S1 Video in supplementary material”. The movie shows the simulation of the diffusion of NS5A on the ER surface, (8). At the beginning, the NS5A concentration is small within the (bleached) FRAP ROI F. During the simulation, the diffusion of NS5A enhances the FRAP ROI concentration again. (The complete equilibrium is not reached within the time which corresponds to the time of the FRAP experiments as within the experimental case [34]). Red indicates high NS5A concentration, blue low concentration. Right hand side: zoom of the zone around the FRAP ROI.

**Figure 5:**
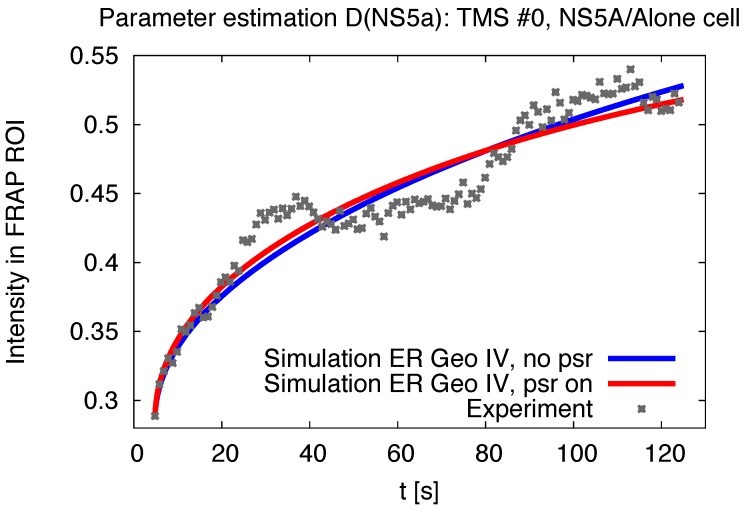
FRAP region intensity evaluation: experiment and simulation (computed with I of (12)), example of NS5A/Alone cell case. The curves depict the uprise of the concentration within the (bleached) FRAP ROI F for the in vitro and the in silico case. The in silico case inherits two different ways of theoretical description: The case $r_{p}^{A}=0$ (“no psr”) neglects the measurement process induced signal reduction within (8), whereas the other case incorporates the afore estimated non-zero value of $r_{p}^{A}$ (“psr on”, value cf. (11)). The in silico curves are that curves which arise for the estimated optimal value of $D_{\mathrm{ns}5a}^{A}$ for TMS # 0 of the NS5A/Alone cell case adapted to the reconstructed ER geometry $E_{4}$.

**Figure 6:**
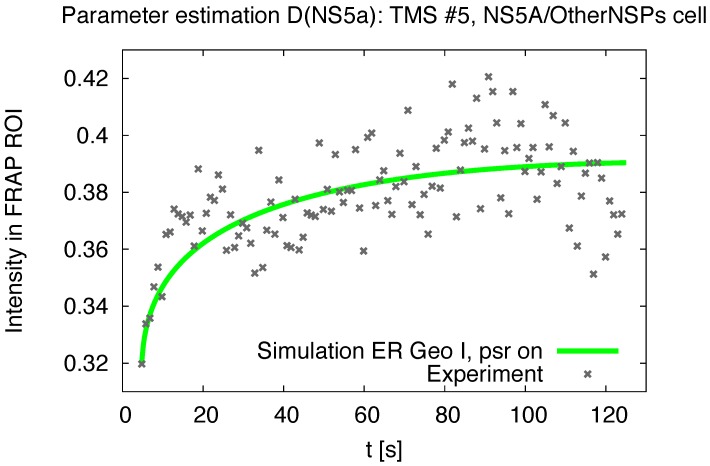
FRAP region intensity evaluation: experiment and simulation (computed with I of (12)), example of NS5A/OtherNSPs cell case. The curves depict the uprise of the concentration within the (bleached) FRAP ROI F for the in vitro and the in silico case. The in silico case inherits only the $r_{p}^{N}\neq 0$ case ("psr on", value cf. (11)) which models the measurement process induced signal reduction within (8). The in silico curves are that curves which arise for the estimated optimal value of $D_{\mathrm{ns}5a}^{N}$ for the time series # 5 of the NS5A/OtherNSPs cell case adapted to the reconstructed ER geometry $E_{1}$.

Our diffusion model of NS5A on the ER surface as derived in Section 2.4 was applied to the experimental data based reconstructed ER geometries which we explained in Section 2.6. A screenshot of a single FRAP experiment simulation (8) for one of the combinations (time series—geometric setup) is shown in Figure 4. Figure 4 is a screenshot from supplemental movie “S1 Video in supplementary material”. Figure 5 and Figure 6 depict typical comparison curves of experiment and simulation, i.e., of FRAP ROI intensities (as explained in Section 2.7). Throughout this paper, we use for figures the notations: “TMS”—“time series”, “geo(m)”—geometry, “psr”—“pseudo reaction”.

### 3.2. Estimation of the NS5A Diffusion Constant

The above-mentioned sPDE techniques were applied to estimate the NS5A diffusion constant on the ER surface for the experimental time series of both NS5A/Alone and NS5A/OtherNSPs Huh7 cells.

In detail, the single simulations were performed as explained before in Section 3.1. The simulation intensity of the FRAP region (12) of these single in silico processes was then fitted to the experimental time series to obtain the optimal diffusion constant for each single combination of time series and ER geometry as explained in Section 2.7, for examples, cf. Figure 5 and Figure 6.

The final diffusion constant was calculated as the average of the estimated diffusion constants of all combinations time series—geometric setups (with the methods described in Section 2.7). Before reporting the entire final values, we want to explain the investigations we performed to ensure the validity of our results.

### 3.3. Influence of Geometry and Time Series

**Figure 7:**
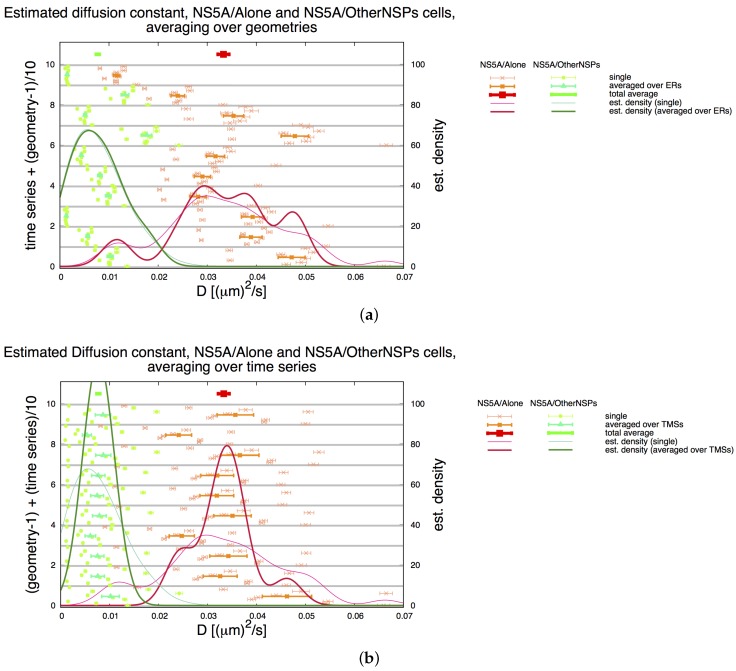
Averages for NS5A diffusion constant $D_{\mathrm{ns}5a}$ estimation on the ER surface as described in Section 2.2 and Section 3.3: Analyzed separately for NS5A/Alone (red—$D_{\mathrm{ns}5a}^{A}$) and NS5A/OtherNSPs (green—$D_{\mathrm{ns}5a}^{N}$). Thin points and error bars correspond for (**a**,**b**) to estimated values for $D_{\mathrm{ns}5a}$ (shown on x axis) for the combination of single TMS with single geometries (indicated on the left y axis, note different combinations for geometry and TMS). Aggregating over all TMS and ER geometries yields distributions (thin continuous lines, scale shown on right y axis) which are identical in both cases. (**a**) Each “row” corresponds to one time series combined with all ER geometries. (For example, the left y axis value 2.5 corresponds to the combination of time series #2 and ER geometry $E_{6}$). Half thick symbols (shown in the middle of each time series region) correspond to the averaged values over all geometries ${\bar{D}}_{\mathrm{ns}5a}{|}_{G}$ for the respective TMS. Aggregating these averages over all TMS yields distributions (thick continuous lines, scale shown on right y axis). Thick symbols (shown on top) correspond to the averaged values ${\bar{D}}_{\mathrm{ns}5a}$. (**b**) Each “row” corresponds to one ER geometry combined with all TMS. (For example, the left y value 2.5 corresponds to ER geometry $E_{3}$ and TMS # 5). Half thick symbols (shown in the middle of each ER geometry region) correspond to the averaged values over all TMS ${\bar{D}}_{\mathrm{ns}5a}{|}_{T}$ for the respective ER geometry as reported in Section 2.6. Aggregating these averages over all ER geometries yields distributions (thick continuous lines, scale shown on right y axis). Thick symbols (shown on top) correspond to the averaged values ${\bar{D}}_{\mathrm{ns}5a}$. Note: The total averages are identical for (**a**,**b**) and are reported in Table 1.

To minimize the risk of artificial errors caused by the independent geometric setups, we tested intensively the influence of the time series and of the geometric setups on the final averaged diffusion constant. We found only a minor influence of the geometric setups (for both cell types). Figure 7 shows the distributions of the single and averaged values. The thick distribution curves of Figure 7a reflect the results for the single geometries as averaged over all time series, whereas the thick distribution curves of Figure 7b reflect the results for single time series averaged over all geometries. The distribution curves show that the geometric setups have only a minor influence in comparison to the time series. Therefore, the use of other realistic geometric setups from independent ER staining data [5] instead of the non available original ones of the TMS data [34] seems to be justified.

### 3.4. Refinement Stability

The numerical stability of the results averaged over the geometric setups and the time series for the respective two cell types concerning the refinement level was intensively tested and showed sufficiently converging results for one-fold spatial refinement based sPDE evaluations. For details, cf. Appendix E.

### 3.5. Influence of the Measurement Process

The diffusion constant estimations were also done for zero pseudo reaction demonstrating the importance of the consideration of the measurement based intensity reduction. Only the NS5A/Alone cell case allowed for the derivation of reliable results for $r_{p}=0$. For the detailed results, we refer to Appendix D. Numerically, we also tested for the dependence of $D_{\mathrm{ns}5a}$ on $r_{p}$ (covering also non-biophysical values) and found an excellent linear agreement, cf. Appendix F.

### 3.6. Comparative 2D Simulations

Comparative computations were done for a continuum model based on a planar 2D geometry with circular F to test for the influence of the curved ER manifold on the dynamics. This simulation type corresponds to the classical type of FRAP modeling, because the geometric structure is not resolved in this simplified case. The fit for each one of the 20 time series and the averaging for NS5A/Alone and NS5A/OtherNSPs cells showed a significant difference compared to the case when the ER structure is resolved. This demonstrates the importance of the use of a correct model [71].

**Figure 8:**
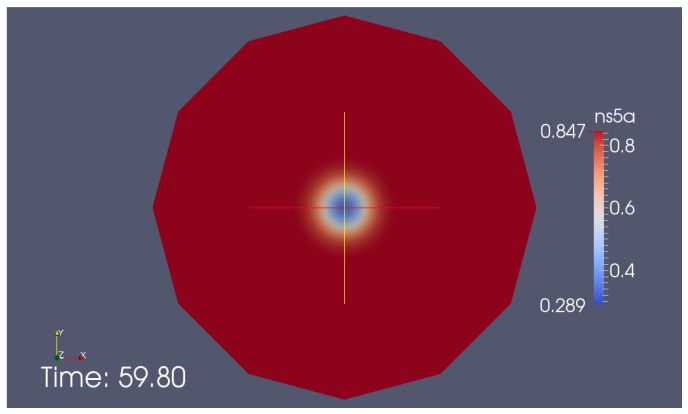
Classical 2D simulation of the FRAP process: Screenshot of supplemental movie “S3 Video in supplementary material”. Simulation of FRAP experiment on simple 2D planar continuum geometry. (Red high concentration, blue low concentration). At the beginning, the bleached region has low concentration, but the 2D diffusion refills it again during the process, i.e. the color shifts slowly to red again because from the high concentration unbleached region around, fluorescating NS5A is diffusing inside. The ER structure is neglected.

Figure 8 depicts a screenshot of a simulation at the 2D plane geometry referring to supplemental movie “S3 Video in supplementary material”. In Appendix C we show the distribution of the estimated diffusion constant corresponding to the single time series and the averaged results of both cell types (in a similar way as performed in Figure 7 for the ER surface case).

### 3.7. Final Averaged Results

The detailed investigations we performed and which we describe previously demonstrated: We have derived stable averaged results of the optimized NS5A diffusion constant on the ER surface for the NS5A/Alone and NS5A/OtherNSPs cell types based on 10 realistic reconstructed ER scenarios combined with 10 respective experimental FRAP time series.

The final averaged results for both cell types are shown in Table 1 for the ER manifold surface and for the 2D planar case.

The diffusion constant for the NS5A/Alone cells was approximately 4-fold larger than that of the NS5A/OtherNSPs cells (for both geometry types, i.e., ER surface and 2D planar geometry case).

Hence, the estimated value for the NS5A/OtherNSPs is significantly smaller then that one of NS5A/Alone and indicates that in the presence of other NSPs, the NS5A mobility is substantially reduced, presumably due to a higher amount of NS5A clustered to DMVs or membranous web regions.

The parameter estimations based on simplified 2D planar geometry, rather than the ER surface setup, caused a decrease of the diffusion constant values by a factor of approximately 2 (for both cell types). Thus, geometric simplifications as used often within simulations of biophysical processes change the results significantly.

## 4. Discussion

We derived values for the NS5A diffusion constant of two experimentally important cell types, namely NS5A/Alone and NS5A/OtherNSPs cells.

### 4.1. Interpretation of the Diffusion Constant Values

The results show a 4-fold decrease in the diffusion constant observed within NS5A/OtherNSPs compared to NS5A/Alone cells (cf. Table 1). This decrease in the diffusion constant indicates a substantial effective reduction of the mobility of NS5A when the protein is expressed together with other NSPs in comparison to the situation when NS5A is expressed alone. Since the relation between those NS5A proteins clustering together to form DMVs or membranous web regions and those which remain freely diffusing on the ER surface has not been quantified experimentally to date, we assume that the diffusion constant of the freely diffusing NS5A species is identical for both cell lines. Therefore, the likely reason for the decreased effective diffusion constant observed when other NSPs are present is the presence of other NSPs enhancing the accumulation/clustering rate of NS5A into DMVs or membranous web regions. The clustered NS5A protein species have greatly reduced mobility. If more NS5A proteins cluster, the overall effective diffusion constant is reduced. This occurs when other NSPs are expressed (as in the case of our NS5A/OtherNSPs cell type) and not when only NS5A is expressed alone (which is valid for our NS5A/Alone cell type).

We want to analyze this property quantitatively based upon our mathematical model: According to the model expressed in Section 2.4, $D^{-}$ identifies the diffusion coefficient of the non-clustered (−) fraction of NS5A. Since this species is freely diffusing along the ER surface, we can assume that this constant is identical in both cell types, i.e., $D_{A}^{\left(\pm \right)}=D_{N}^{\left(\pm \right)}$. The four-fold increase of the effective diffusion constant once NS5A is expressed alone indicates obviously that less NS5A belongs to the clustered (+) fraction of NS5A. Even though some fraction of NS5A clusters also for the NS5A/Alone case, this fraction is substantially reduced in comparison to the NS5A/OtherNSPs case. Therefore, even though $c_{A}^{\left(+\right)}\neq 0$, we conclude $c_{A}^{\left(+\right)}\ll c_{N}^{\left(+\right)}$. Therefore, we may consider the limiting case $c_{A}^{\left(+\right)}⟶0$ as a first order approximation for mathematical-quantitative consideration. Hence, we want to consider the most extreme case assuming for a moment that *all* NS5A do not cluster if NS5A is expressed alone. Of course, this is likely not the actual case, but the strongly enhanced portion of NS5A which is not clustering in the NS5A/Alone cell case compared to the NS5A/OtherNSPs case allows to consider this extreme approximation as some sort of first order approximation. The first-order hypothesis that all NS5A are not clustering for the NS5A/Alone case indicates that $k_{\mathrm{on}}^{A}⟶0$ within (2). Since we assume that the diffusion constant of both species of NS5A is the same for both cell types, $D_{A}^{\left(\pm \right)}=D_{N}^{\left(\pm \right)}$, the difference for the both cell types originates within the different binding rates, $k_{\mathrm{on}}^{A}\neq k_{\mathrm{on}}^{N}$ respectively $k_{\mathrm{off}}^{A}\neq k_{\mathrm{off}}^{N}$. Within our modeling framework, this hypothesis corresponds to differences in $D_{\mathrm{ns}5a}$ from Equation (7): Under the assumptions (3), the fitted results to ratio $k_{\mathrm{on}}^{N}/k_{\mathrm{off}}^{N}=3$ for long times for the limit case $k_{\mathrm{on}}^{A}≃0$. That indicates a ratio of 1:3 for $c_{N}^{\left(-\right)}$ to $c_{N}^{\left(+\right)}$ due to (4), i.e., for free versus clustered species for the NS5A/OtherNSPs case within our first order approximation. This means that in the NS5A/OtherNSPs case, an appreciable amount of NS5A molecules are clustered in membranous web regions / DMVs, and thus relatively immobile.

The diffusion of NS5A species on the ER surface that are not clustered into DMVs/membranous webs are likely not completely “free” on the ER surface due to interactions with a huge possible number of host interacting proteins (about 130 [72]). However, our results indicate that the most important obstruction of the NS5A movement originate from its clustering to DMVs or web regions when additional NSPs are present. This would account for the 4-fold diffusion constant decrease once other NSPs are expressed.

To the best of our knowledge, applying spatio-temporal modeling to biological data has not been previously performed in this manner and forms basis for further detailed investigation and refinement using subsequent models [27].

### 4.2. The Context of Spatial HCV Models

We are developing spatio-temporal resolved models of the HCV replication cycle within single liver-derived cells. In our previously published paper [27], we developed spatially resolved models of the vRNA cycle. These models considered the major components of the vRNA cycle, namely vRNA, NSPs and a host factor. The model was qualitative rather then quantitative due to a lack of experimentally-derived parameters. The diffusion constant for the NS5A/Alone case which we derived in this study is a candidate for the class of diffusion-reaction models of HCV replication [27]. Future work will ask for the determination of the diffusion constants of the other components of the vRNA replication cycle.

### 4.3. Related Work

The control computations using a 2D continuum model gave values which for the two cell types were about a factor 2 smaller than for the ER surface simulations. The differences between ER manifold sPDE and 2D continuum PDE results are in agreement with observations based on particle based PDE evaluations [73,74]. Only few publications deal with the quantitative analysis of spatio-temporal properties of virus proteins [31,75] at all. Firm values for viral protein diffusion constants have not been reported in the literature. For metabolism proteins and other physiologic ingredients, some evaluations exist, cf. e.g., [73,74,76,77,78,79,80,81,82,83,84,85,86,87]. Besides the studies published within [73,74], these approaches do not take into account the detailed structure of the ER. In most cases, the PDE evaluations are based on simplified techniques. Advanced numerical methods have been applied only in very few cases within the field of cellular simulations at the ER [88] yet.

## 5. Conclusions

The estimation of biophysically meaningful results for the diffusion constant of the important NS5A viral protein on the curved ER surface manifold is an intellectually-stimulating contribution to the young field of spatio-temporal resolved research within computational virology. For the first time, the derived results give a quantitative biophysical description of the movement properties of a crucial viral protein related to various steps of virus replication—indeed, we believe the parameter estimated by us represents the first quantitative description of the movement characteristics of any viral component at an (intra)cellular level. These results are intended to enter spatio-temporal resolved biophysical models of HCV replication in the type we have presented previously in our model paper [27], and, later, the techniques can be applied to similar systems [2,3,4,11,12,13,14,15,16,17,18,19,20,89] including non-viral processes [88], e.g., metabolic protein [73,74,76,77,78,79,80,81,82,83,84,85,86,87] dynamics.

Our novel approach to introduce spatio-temporal resolved simulation techniques into computational virology paves a way for previously unexplored detailed biophysical understanding of virus replication dynamics, for example to unveil the relationship of form and function, as we have demonstrated already within our former paper [27], or to reveal areas of the virus life cycle amenable to novel antiviral intervention that conventional biology may miss e.g., spatial dependence of virus-encoded factors within specfic intracellular regions. In the future, this avenue of research has the possibility of substantially impacting our understanding of complex biological systems.

## Figures and Tables

**Table 1:** Averaged final NS5A diffusion constant ${\bar{D}}_{\mathrm{ns}5a}$ as described in Section 3.7. The final values are computed by means of the averaging process of the single results (as described in Section 2.7) which was shown graphically within Figure 7. We give results for the case of the use of the ER geometry setups as described in Section 3.1 and Section 3.2, but also for a simplified classical 2D planar consideration, cf. Section 3.6.

Geoms	$D\left[{\left(\mu m\right)}^{2}/s\right]$	$\sigma \left[{\left(\mu m\right)}^{2}/s\right]$
	NS5A/Alone cell type	
plane 2D	0.014815	0.001546
ER surface	0.033307	0.001142
	NS5A/OtherNSPs cell type	
plane 2D	0.003873	0.000695
ER surface	0.007696	0.000353

**Figure A1:**
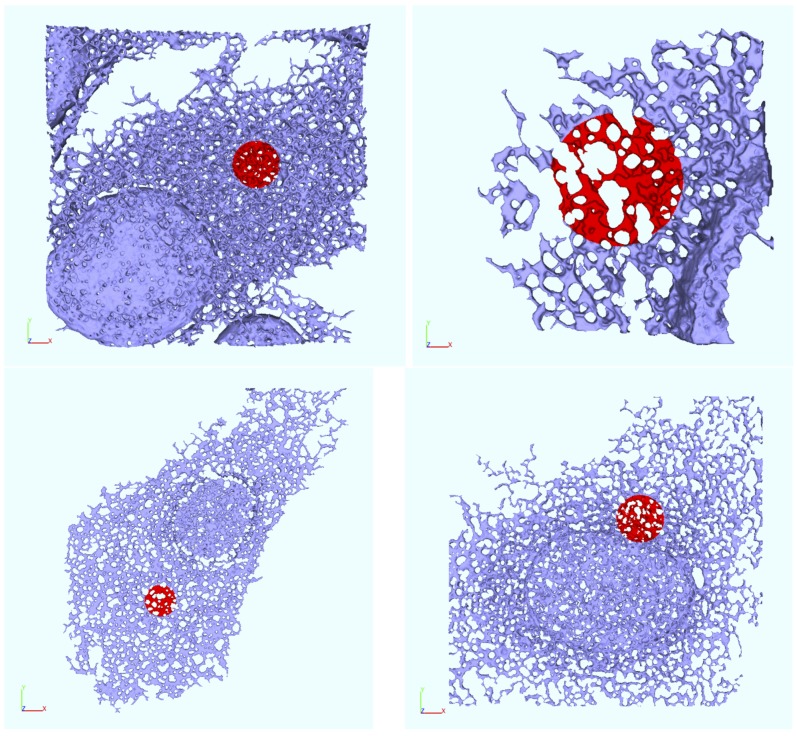
The reconstructed ER geometries $E_{i}$, i=2,3,4,5 and exemplar FRAP regions. FRAP ROIs indicated in red, covering a surface of about 38 μm${}^{2}$ enabling the reader to estimate the total size of the ERs.

**Figure A2:**
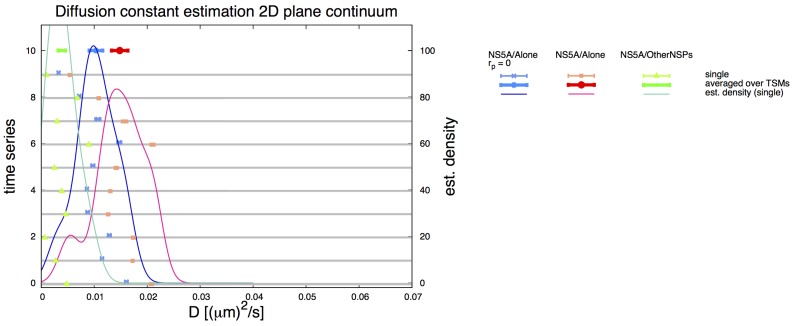
Classical FRAP analysis based upon the 2D computations explained in Section 3.6: Averages for diffusion constant estimation $D_{\mathrm{ns}5a}$—analyzed separately for NS5A/Alone (red—$D_{\mathrm{ns}5a}^{A}$), NS5A/OtherNSPs (green—$D_{\mathrm{ns}5a}^{N}$) as well as for NS5A/Alone setting $r_{p}=0$ (blue—$D_{\mathrm{ns}5a}^{A}{|}_{r_{p}=0}$), i.e., assuming that the measurement process induces no intensity reduction. Thin points and error bars correspond to estimated values for $D_{\mathrm{ns}5a}$ (shown on x axis) for a single TMS (indicated on the left y axis). Aggregating over all TMS yields distributions (continuous lines, scale shown on right y axis). Thick symbols (shown on top) correspond to the averaged values ${\bar{D}}_{\mathrm{ns}5a}$ reported in Table 1 for the 2D planar case and in Table A3 for the 2D planar case using $r_{p}^{A}=0$ for the NS5A/Alone cell case.

**Figure A3:**
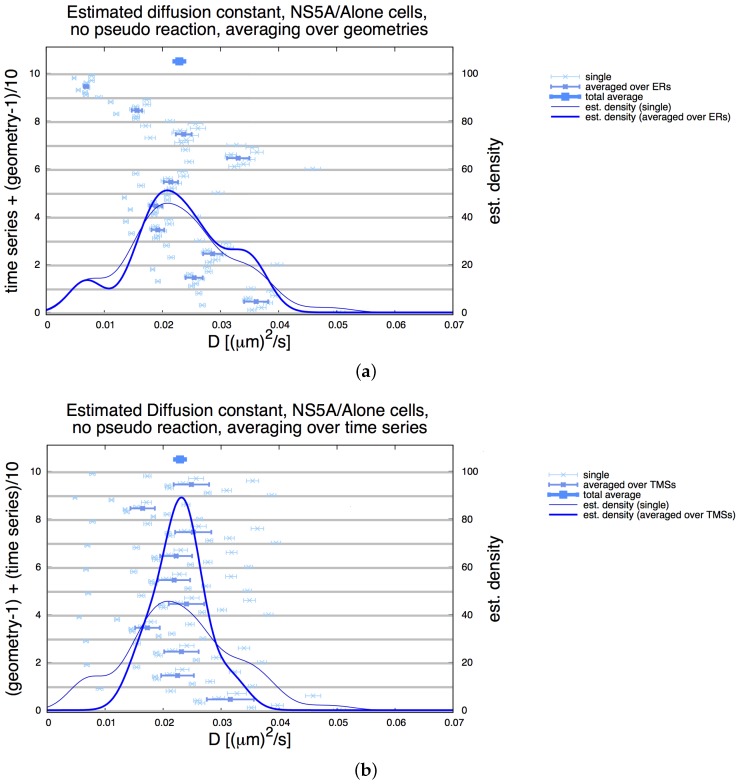
Averages for NS5A diffusion constant $D_{\mathrm{ns}5a}^{A}$ estimation on the ER surface as described in Section 2.2 and Section 3.3 for the NS5A/Alone cell case, neglecting the measurement process induced signal reduction, i.e., setting $r_{p}^{A}=0$ in (8). Simular analysis as in Figure 7. Thin points and error bars correspond for (**a**,**b**) to estimated values for $D_{\mathrm{ns}5a}$ (shown on x axis) for the combination of single TMS with single geometries (indicated on the left y axis, note different combinations for geometry and TMS). Aggregating over all TMS and ER geometries yields distributions (thin continuous lines, scale shown on right y axis) which are identical in both cases. (**a**) Each “row” corresponds to one time series combined with all ER geometries. (For example, the left y axis value 2.5 corresponds to the combination of TMS # 2 and ER geometry $E_{6}$). Half thick symbols (shown in the middle of each time series region) correspond to the averaged values over all geometries ${\bar{D}}_{\mathrm{ns}5a}{|}_{G}$ for the respective TMS. Aggregating these averages over all TMS yields distributions (thick continuous lines, scale shown on right y axis). Thick symbols (shown on top) correspond to the averaged values ${\bar{D}}_{\mathrm{ns}5a}$. (**a**) Each “row” corresponds to one ER geometry combined with all TMS. (For example, the left y value 2.5 corresponds to ER geometry $E_{3}$ and TMS # 5). Half thick symbols (shown in the middle of each ER geometry region) correspond to the averaged values over all TMS ${\bar{D}}_{\mathrm{ns}5a}{|}_{T}$ for the respective ER geometry as reported in Section 2.6. Aggregating these averages over all ER geometries yields distributions (thick continuous lines, scale shown on right y axis). Thick symbols (shown on top) correspond to the averaged values ${\bar{D}}_{\mathrm{ns}5a}^{A}$. Note: The total averages are identical for (**a**,**b**) and are reported in Table A3.

**Figure A4:**
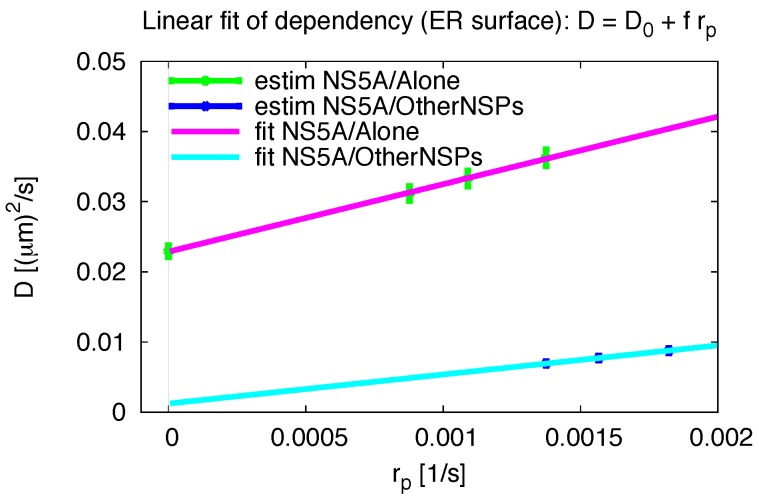
Dependency diffusion constant of NS5A on ER surface on pseudo reaction constant, linear fit of (A1) for numerical stability test reasons. “estim”: estimated values, “fit”: linear fit.

**Figure A5:**
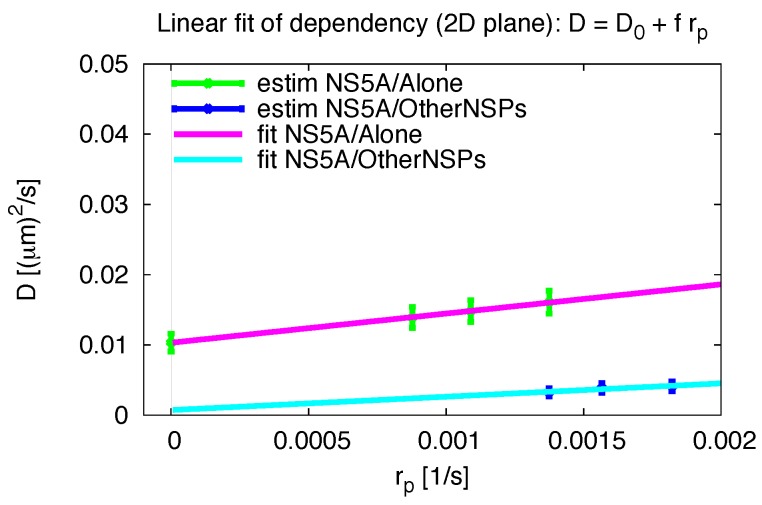
Dependency diffusion constant on pseudo reaction constant, linear fit of (A1) for 2D planar geometry. “estim”: estimated values, “fit”: linear fit.

**Figure A6:**
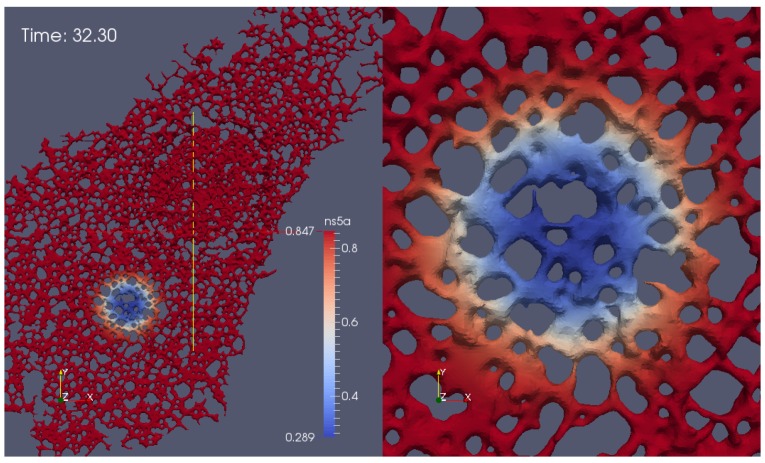
Screenshot of NS5A FRAP experiment simulation on ER surface, Geometry IV (suppl. movie, “S2 Video in supplementary material”).

## Supplementary Materials

The following are available online at www.mdpi.com/1999-4915/10/1/28/s1. Supplemental Movie S1 Video: Movie of FRAP simulation at ER geometry I, Supplemental Movie S2 Video: Movie of FRAP simulation at ER geometry IV, Supplemental Movie S3 Video: Movie of classical FRAP simulation at 2D continuum plane. (cf. also the Appendix A.1, Appendix A.2 and Appendix A.3 for brief descriptions of the simulation movies).

## Abbreviations

The following abbreviations are used in this manuscript:

FRAP	Fluorescence recovery after photobleaching
FLIP	Fluorescence loss in photobleaching
ROI	region of interest
HCV	Hepatitis C virus
vRNA	viral RNA
NSP	non structural viral protein
NS5A	HCV non structural protein number 5
SP	structural protein
DMV	Double membrane vesicle
TMS	time series
ER	Endoplasmatic Reticulum
ODE	Ordinary Differential Equation
PDE	Partial Differential Equation
sPDE	surface Partial Differential Equation
FV	Finite Volumes
MG	Multi Grid
GMG	Geometric Multi Grid
UG4	Unstructured Grids version 4 [50,51]
NeuRA2	Neuron reconstruction algorithm, version 2.3 [35,68]
geo(m)	geometry
psr	pseudo reaction

## Appendix A. Supplemental Movies, Short Description

### Appendix A.1. S1 Video: Movie of FRAP Simulation at ER Geometry I

Simulation of NS5A FRAP experiment simulation at realistic reconstructed ER geometry I (computed by means of surface PDE description of NS5A diffusion (8) with UG4).

### Appendix A.2. S2 Video: Movie of FRAP Simulation at ER Geometry IV

Simulation of NS5A FRAP experiment simulation at realistic reconstructed ER geometry IV (computed by means of surface PDE description of NS5A diffusion (8) with UG4).

### Appendix A.3. S3 Video: Movie of Classical FRAP Simulation at 2D Continuum Plane

Simulation of NS5A FRAP experiment simulation at 2D continuum plane (computed by means of PDE description of NS5A diffusion with UG4, $\Delta_{\left(T\right)}\to \Delta$, i.e., “trivial” 2D diffusion instead of manifold surface diffusion, use of “common” 2D Laplace operator $\Delta =\partial^{2}/\partial x^{2}+\partial^{2}/\partial y^{2}$ in (8)).

## Appendix B. Realistic Reconstructed ER Geometries—Further Details

Figure A1 depicts the basic ER geometries $E_{i}$, i=2,3,4,5, i.e., all ER geeometries with one FRAP ROI example (ER geometry $E_{1}$ is shown in Figure 3).

Table A1 depicts the number of the degrees of freedom (DoF) and the number of the basic elements (triangles) of the ER geometries corresponding to the different subdomains. Roughly, the number increases about a factor 4 for each refinement level. For more details, cf. [35].

ER geometries 6–10 harbor the same number of DoFs and faces, however slightly different numbers within F/U caused by the shift of the FRAP ROI.

## Appendix C. Simple Planar 2D Geometry and Simulations—Details

Table A2 depicts the number of DoFs for the simple planar 2D geometry based classical 2D FRAP modeling which is used for comparison computations, i.e., when no ER structure is taken into account. cf. Section 3.6.

Figure A2 depicts the distribution of the estimated diffusion constant corresponding to the single time series and the averaged results of both cell types.

## Appendix D. Neglecting the Measurement Process Induced Intensity Reduction

For the NS5A/Alone cell case, we estimated the diffusion constant also setting $r_{p}^{A}=0$ within the basic sPDE (8). (For the NS5A/OtherNSPs case, this was not possible reliably, i.e., using $r_{p}^{N}=0$ did not allow to estimate reliable diffusion constants).

Figure A2 inherits the single (i.e., for each time series) estimated diffusion constant values, their distribution and the averaged diffusion constants evaluated for the 2D case. Figure A3 shows the single and averaged results and the distributions for the computations on the ER surface. Table A3 depicts the final averaged values for the case $r_{p}^{A}=0$ for the NS5A/Alone case, for the ER surface based computations and for the classical 2D plane based computations.

**Table A1:** DoF number and total face number of the ER geometries at level L, extracted from our former paper [35].

Geo	L	DoF E	DoF U	DoF F	Faces
$E_{1}$	0	815,111	794,128	20,983	1,636,803
	1	3,270,924	3,187,566	83,358	6,547,212
	2	13,092,959	12,761,035	331,924	26,188,848
$E_{2}$	0	1,212,622	1,174,204	38,418	2,430,181
	1	4,861,079	4,708,431	152,648	9,720,724
	2	19,448,536	18,840,730	607,806	38,882,896
$E_{3}$	0	170,209	140,022	30,187	340,108
	1	680,786	560,740	120,046	1,360,432
	2	2,722,264	2,243,664	478,600	5,441,728
$E_{4}$	0	601,706	591,336	10,370	1,208,661
	1	2,414,802	2,373,711	41,091	4,834,644
	2	9,666,977	9,503,511	163,466	19,338,576
$E_{5}$	0	728,636	699,338	29,298	1,463,597
	1	2,924,907	2,808,345	116,562	5,854,388
	2	11,708,240	11,243,894	464,346	23,417,552

**Table A2:** DoF number and total face number of the simple planar 2D geometry at level L, extracted from our former paper [35].

L	DoF E	DoF U	DoF F	faces
0	37	24	13	36
1	133	96	37	144
2	505	384	121	576
3	1,969	1,536	433	2,304
4	7,777	6,144	1,633	9,216
5	30,913	24,576	6,337	36,864
6	123,265	98,304	24,961	147,456
7	492,289	393,216	99,073	589,824
8	1,967,617	1,572,864	394,753	2,359,296

## Appendix E. Refinement Stability

In our former paper [35], we investigated the refinement stability of single sPDE computations for heuristic diffusion constant values (setting $r_{p}=0$). We tested the stability of the temporal evolution of the FRAP ROI integrals I(t), (12), under variation of spatial and temporal refinement. Therefore, we evaluated the relative differences under spatial level resp. time step size refinement.

Here, we analyze the refinement stability of the estimated averaged diffusion constant. As shown in our paper [35], the influence of the refinement stability increases when the diffusion constant decreases. Since the pseudo reaction does not depend on spatial refinement, we only needed to check for the case $r_{p}=0$ in (8) in principle. Therefore, we check the NS5A/Alone cell case without pseudo reaction ($r_{p}^{A}=0$). (The corresponding single and averaged results were discussed within Appendix D afore). Since the NS5A/OtherNSPs cell case did not allow for a parameter estimation without incorporating the pseudo reaction, we perform the check in this case with pseudo reaction ($r_{p}^{N}\neq 0$)).

In order to save computation time, we omitted to perform the level 2 computations for the second location of the FRAP ROIs and compared the results for one FRAP ROI per reconstructed ER geometry. Table A4 presents the investigations of the spatial refinement stability of the averaged Gauss-Newton results for the NS5A/Alone cell case and for the NS5A/OtherNSPs cell case (using respectively only one FRAP ROI per geometry for simplicity). Our standard time step size 0.1 s has only minor influence on the numeric error compared to the spatial refinement influence [35]. Hence, there was no need to check the stability of the averaged results concerning the time step size refinement. Finally we omitted extended refinement stability checks for NS5A/OtherNSPs cells combined with the planar 2D geometry since no additional insight would have been gained.

The averaged values considering only 5 geometric setups (Table A4) are negligible different (3%) from the final values averaged over all 10 geometric setups, Table 1 (for level 1 computations).

Based on the comparisons shown in Table A4, we conclude that level 1 is sufficient for the ER computations and level 8 for the 2D case computations.

**Table A3:** Averaged final NS5A diffusion constant ${\bar{D}}_{\mathrm{ns}5a}^{A}$ (for the NS5A/Alone cell case) as described in Section 3.7, but assuming vanishing signal reduction due to the measurement process, i.e., using $r_{p}^{A}=0$ in (8). The final values are computed by means of the averaging process of the single results (as described in Section 2.7) which is shown graphically within Figure A3. We give results for the case of the use of the ER geometry setups as described in Section 3.1 and Section 3.2, but also for a simplified classical 2D planar consideration, cf. Section 3.6. Values only given for the NS5A/Alone case, since reliable estimations were not possible for the NS5A/OtherNSPs case once we used $r_{p}^{N}=0$.

Geoms	$D\left[{\left(\mu m\right)}^{2}/s\right]$	$\sigma \left[{\left(\mu m\right)}^{2}/s\right]$
	$r_{p}^{A}=0$ **, NS5A/Alone cells**	
plane 2D	0.010328	0.001274
ER surface	0.022915	0.000887

**Table A4:** Refinement stability: Averaged NS5A diffusion constant for the simplified planar 2D case and the ER surface manifold computations (using only one FRAP ROI per ER geometry. Nota bene: the afore reported final results cover all geometric setups, i.e., the final results reported in Table 1 are based upon the use of two FRAP ROIs per reconstructed ER geometry). Evaluation for different spatial refinement levels R of base geometry and relative change C in comparison to level before in percentage. Values for the NS5A/Alone cell case without pseudo reaction, i.e., $r_{p}^{A}=0$ in (8) and for the NS5A/OtherNSPs cell case with pseudo reaction, $r_{p}^{N}\neq 0$.

Geometries	R	$D\left[{\left(\mu m\right)}^{2}/s\right]$	$\sigma \left[{\left(\mu m\right)}^{2}/s\right]$	C [%]
**NS5A/Alone Cells**, $r_{p}^{A}=0$				
2D planar	5	0.012133	0.001415	—
	6	0.011093	0.001334	9.378
	7	0.010582	0.001294	4.832
	8	0.010328	0.001274	2.452
5 ERs	1	0.023701	0.001309	—
	2	0.023239	0.001288	1.989
**NS5A/OtherNSPs Cells**, $r_{p}^{N}\neq 0$				
5 ERs	1	0.007837	0.000513	—
	2	0.007602	0.000493	2.996

## Appendix F. Variation of Pseudo Reaction

We tested for the influence of the numerical variation of the pseudo reaction constant on the final result of the diffusion constant, i.e., we analyze how the final diffusion constant changes under variation of the value of $r_{p}$ in our “master sPDE” (8).

Therefore, we estimated the diffusion constant $D=D_{\mathrm{ns}5a}$ also for (non-biophysical) values of $r_{p}$ in the region of the biophysical values. Hence, the estimations of $D_{\mathrm{ns}5a}$ were performed not only for the estimated values of $r_{p}$, but also for other ones close to them to test for the dependency.

Due to the smallness of the scale of $r_{p}$—$O\left(10^{-3}\right)$—the variation of *D* can be very well approximated by a linear fit, since higher terms in the Taylor approximation are negligible. Using the linear regression ansatz

(A1) $$D\left(r_{p}\right)=D_{0}+fr_{p}+O\left(r_{p}^{2}\right)$$

we derived the factors $D_{0},f$. A graphical representation of the fit for the ER surface computations is given in Figure A4. Figure A5 depicts the variation for the 2D case in graphical representation. Table A5 shows the numerical coefficients of (A1) for the ER surface calculations. Table A6 depicts the coefficients for the 2D case. The graphs, coefficients of the fits and the fit procedure are shown here in the appendices since this investigation is not of biophysical interest, but moreover serves as an additional numerical stability check. The quality of the linear fits, i.e., the excellent linear agreement, can be considered as a further hint of the stability of our results.

## Appendix G. Additional Simulation Screenshot

Whereas Figure 4 depicts a screenshot of the simulations at ER geometry I, Figure A6 shows an additional simulation screenshot of the ER computations, namely at ER geometry IV. The corresponding movie is attached as supplemental movie “S2 Video in supplementary material”.

**Table A5:** Fit parameters in (A1) of linear regression dependency of diffusion constant value on pseudo reaction constant, on ER surface.

Cells	$D_{0}\left[{\left(\mu m\right)}^{2}/s\right]$	$f\left[{\left(\mu m\right)}^{2}\right]$
NS5A/Alone	0.0228726	9.62539
NS5A/OtherNSPs	0.0012175	4.14657

**Table A6:** Fit parameters in (A1) of linear regression dependency of diffusion constant value on pseudo reaction constant on 2D planar geometry.

Cells	$D_{0}\left[{\left(\mu m\right)}^{2}/s\right]$	$f\left[{\left(\mu m\right)}^{2}\right]$
NS5A/Alone	0.0103104	4.15337
NS5A/OtherNSPs	0.0007236	1.89964
